# Internal Pressure–Temperature Coupling Analysis Method for Thermal Decomposition of GFRP Composites Based on the Overlapping Elements Method

**DOI:** 10.3390/ma17030756

**Published:** 2024-02-04

**Authors:** Han Li, Peng Wei, Xuefei Han, Jiawei Li

**Affiliations:** 1 Science and Technology Innovation Research Institute, Civil Aviation University of China, Tianjin 300300, China; 2 College of Safety Science and Engineering, Civil Aviation University of China, Tianjin 300300, China; pengw0715@163.com (P.W.); ljw00828@163.com (J.L.); 3 Avic Beijing Keeven Aviation Instrument Co., Ltd., Beijing 101300, China; hanxuefei185@163.com

**Keywords:** inter pressure-temperature coupling analysis, thermal decomposition, GFRP, overlapping elements method

## Abstract

A method of internal pressure–temperature coupling analysis for the thermal decomposition of GFRP composites under high-temperature conditions was established, which incorporates coupled calculations of heat transfer equations, the Arrhenius equation, Darcy’s law, and the ideal gas state equation. Using the overlapping mesh method, the coupling calculation of temperature and internal pressure is realized based on the UMATHT and USDFLD user subroutines developed. Specifically, two user subroutines, UMATHT-1 and UMATHT-2, are used to define the heat transfer equation and gas diffusion equation separately. Numerical simulations are conducted to simulate the polymers’ thermal decomposition in high-temperature environments. For glass fiber/vinyl ester composites and glass fiber/phenolic composites, the predicted temperature and pressure values are in good agreement with experimental measurements, and porosity and permeability are then analyzed. Due to the accumulation of thermal decomposition gases, inter-pressure within the material surged and reached a peak value. After that, it began to decrease, but the factors affecting the pressure decrease vary at different positions. Specifically, the pressure closest to the heating surface is influenced by the combined effects of decomposition rate, permeability, and porosity, while the pressure far away from the heating surface is only affected by the initial permeability. The pressure in the intermediate region may be influenced by both increased porosity and initial permeability.

## 1. Introduction

FRP composites are widely used in aerospace manufacturing due to their excellent performance, such as their light weight, high specific strength, and modulus, for applications such as aircraft radar domes and the cabin interior [1,2,3,4]. The Federal Aviation Administration (FAA) has issued corresponding airworthiness requirements for the use of composite materials on aircraft. For example, AC 20-107B [5] proposes that flame resistance and fire protection requirements should be considered in the design of composite structures. This is because they can soften, deform, and thermally decompose at high temperature [6,7,8,9], forming thermal decomposition products such as CO_2_ and CO, which accumulate and generate pressure within the composites, further causing deformation and delamination failure of composite structures. For this issue, it is necessary to study the internal pressure of composites and the behavior of thermal decomposition in fire environments. 

Henderson et al. [10,11] developed a one-dimensional transient thermal response model based on the expansion equation proposed by Buch [12], which takes into account the thermal decomposition of the matrix material, diffusion of decomposed gases, as well as gas flow and accumulation within the material. Using this model, the temperature, pressure, and material expansion deformation of glass-fiber-reinforced phenolic resin were predicted. Tinney [13] proposed a mathematical model for the combustion of small wooden tenons. By using Fourier conduction equation and the first-order Arrhenius equation, the central temperature, weight loss, and internal pressure history were predicted. Dimitrienko [14,15] developed a mathematical model to describe the high-temperature behavior of glass fiber/epoxy composite materials. This model involves thermal properties such as thermal conductivity, density, and porosity. It was found that the occurrence of internal gas pressure and lateral stress under high-temperature heating can cause delamination in the material. Jun Koyanagi [16] established a one-dimensional mathematical model considering thermal conduction, thermal decomposition, gas flow from thermal decomposition, and internal gas pressure. Coupled analysis of heat transfer and gas flow from thermal decomposition was performed using finite difference form. The model was used to predict the internal gas pressure of carbon fiber composite materials under ablation conditions, and predict delamination caused by internal pressure. Sullivan and Salamon [17,18] proposed a three-dimensional thermo-mechanical coupled model based on Henderson’s model. The equations of energy, mass transfer, and momentum were coupled to predict temperature, pore pressure, and stress distribution. However, in their assumptions, gas permeability and porosity were assumed to be constant. These assumptions may have cumulative effects on the accuracy of the numerical results. Looyeh et al. [19] modified the above models and used their numerical solutions to predict the exact values of the elastic parameters of porosity, which were then used to analyze the thermal decomposition process of glass fiber/polyester resin composite plates. Shi Shengbo, Chen Hailong [20,21] performed a volumetric thermo-mechanical-chemical fully coupled calculation on high-silica/phenolic materials under ablation conditions using COMSOL software. They obtained the field of displacement, temperature, pressure, and resin residue rate of high-silica/phenolic composite materials under one-sided radiant heat flux load.

However, a three-dimensional mathematical model considering thermal conduction, thermal decomposition, gas flow from thermal decomposition, and internal gas pressure has not been found. In the present study, numerical calculations of the thermal decomposition process of GFRP materials and the diffusion process of pyrolysis gas are developed through the UMATHT subroutine. A three-dimensional temperature-internal pressure coupling model for composite materials is established, and a temperature-internal pressure coupling analysis method for polymer-based composite materials is proposed. Factors such as thermal conduction of materials, matrix decomposition, gas diffusion, Darcy’s law, ideal gas equation, convective heat transfer of pyrolysis gas, porosity, permeability, and changes in gas viscosity are considered. It can be used to predict the internal temperature and pressure history of composite materials, and discuss decomposition behavior and the variations in internal pressure.

## 2. Theoretical Model

### 2.1. Three-Dimensional Heat Transfer

The equation for heat transfer of composite materials in a unidirectional flow of gas is presented as Equation (1), according to the following assumptions:

1. No accumulation of decomposition gases in the solid material;
2. No thermo-chemical or volumetric expansion;
3. Thermal equilibrium between the decomposition gases and solid materials.

(1) $$\frac{\partial }{\partial t}\left(\rho h_{s}\right)\;-\;\nabla •\left(k_{1}\frac{\partial T}{\partial x}i\;+{\;k}_{2}\frac{\partial T}{\partial y}j\;+{\;k}_{3}\frac{\partial T}{\partial z}k\right)+\frac{\partial }{\partial x}({\dot{m}}_{g}^{,}h_{g})+Q\frac{\partial_{\rho }}{\partial_{t}}=0$$

The $k_{i}(i=1,2,3)$ in the equation represents the thermal conductivity of the composite material in three coordinate directions; T, t,ρ,${\dot{m}}_{g}^{’}$ are temperature, time, solid density, and mass flux of gases, respectively; $C_{p}$,$C_{\mathrm{pg}}$ are solid specific heat, and specific heat of gases generated from decomposition of resin, respectively; $h_{s}=\int_{T_{0}}^{T}C_{P}\mathrm{dT}$, $h_{g}=\int_{T_{0}}^{T}C_{\mathrm{pg}}\mathrm{dT}$ are solid enthalpy, and enthalpy of gases, respectively; Q is decomposition heat. The first term represents the rate of change of internal energy per unit volume, and the second term represents the conduction flux, where the three mutually perpendicular thermal conductivities $k_{i}(i=1,2,3)$ in this context are functions of temperature and material decomposition stages. The third term represents the convective energy generated by the decomposed gas flowing through the composite material. The last term is the time rate of absorption or generation of heat due to the decomposition reaction.

### 2.2. Gas Diffusion Equation

The gas diffusion equation is introduced as Equation (2), according to the following assumptions:

1. Decomposed gases are in an ideal state
2. Decomposition of gases follows Darcy’s Law
3. Decomposition of gases without further reaction

(2) $$\frac{\partial {\dot{m}}_{g}^{\prime }}{\partial z}=-\frac{\partial \rho }{\partial t}$$

Further derivation of the above equation leads to:

(3) $$\frac{1}{V}\frac{\partial m}{\partial t}+\frac{1}{V}\frac{\partial m_{g}}{\partial t}+\nabla •\left({\dot{m}}_{g}^{\prime }\right)=0$$

The total change in mass of the remaining solid, the gas trapped inside the solid and the gas flowing out of the solid should be zero within the unit control body.

(4) $$P=\frac{m_{g}\mathrm{RT}}{\mathrm{MV}\phi }$$

In the equations above, φ represents the material porosity, and $m_{g}$ represents the gas quality.

(5) $${\dot{m}}_{g}^{\prime }=-\frac{\rho_{g}}{\mu }\gamma_{i}\nabla P$$

The above equation is the three-dimensional Darcy’s law equation in the principal direction, which is combined with the continuity Equation (2) to give the unit gas diffusion equation that can be used to predict gas pressure as follows.

(6) $$-\nabla •\left[\rho_{g}\left(\frac{\gamma_{1}}{\mu }\frac{\partial P}{\partial x}i+\frac{\gamma_{2}}{\mu }\frac{\partial P}{\partial y}j+\frac{\gamma_{3}}{\mu }\frac{\partial P}{\partial z}k\right)\right]+\frac{1}{V}\left(\frac{\partial m}{\partial t}+\frac{\partial m_{g}}{\partial t}\right)=0$$

### 2.3. Decomposition Equation

The decomposition rate equation for the composite is given in the form of the Arrhenius kinetic rate equation in Equation (7) [22].

(7) $$\frac{\partial \rho }{\partial t}=-A\left(\rho_{v}-\rho_{\mathrm{char}}\right){\left[\frac{\rho \;-\;\rho_{\mathrm{char}}}{\rho_{v}\;-\;\rho_{\mathrm{char}}}\right]}^{n}\cdot e^{-E/\mathrm{RT}}$$

In the equations above, $\rho_{char}$ and $\rho_{v}$ represent the density of fully carbonized material and the density of the original material, respectively. A is the activation factor, E is the activation energy, n is the reaction order, and R is the ideal gas constant.

In the thermal decomposition process of composite materials, the extent of thermal decomposition can be represented by the decomposition factor of the composite material. The expression for the decomposition factor (F) in relation to the thermal decomposition rate (S) is as follows.

(8) $$F=\frac{\rho \;-\;\rho_{c}}{\rho_{v}\;-\;\rho_{c}}$$

(9) $$S=-\frac{\Delta \rho }{\rho_{v}\;-\;\rho_{c}}$$

In the above expression, $\rho_{v}$, $\rho_{c}$, and Δρ represent the initial density, carbonized density, and density change of the material, respectively. The decomposition factor (F) ranges from 0 to 1. When F = 1, it indicates that the material has not undergone any decomposition reaction and that it remains in its original state. When F = 0, it indicates that the material has completely thermally decomposed, resulting in carbonized material.

### 2.4. Thermal Boundary Conditions

For the purpose of simulating the temperature–pressure coupled calculation of composite materials under unilaterally heated environments, it is necessary to define the initial conditions and boundary conditions.

Initial boundary conditions:

(10) $$T\;=T_{0},\;\rho =\rho_{v},\;P\;={\;P}_{0},\;{\dot{m}}_{g}^{\prime }=0,\;t\;=0$$

Heating surface boundary conditions:

(11) $$q_{\mathrm{front}}^{\prime \prime }=\left(q_{\mathrm{rad}}^{\prime \prime }-\varepsilon \sigma T_{S}^{4}\right)+h_{\mathrm{front}}\left(T_{\infty }-T_{S}\right)$$

Back thermal boundary conditions:

(12) $$q_{\mathrm{back}}^{\prime \prime }=\varepsilon \sigma \left(T_{\mathrm{back}}^{4}\;-{\;T}_{\infty }^{4}\right)+h_{\mathrm{back}}\left(T_{\mathrm{back}}-\;T_{\infty }\right)$$

In the boundary equations above,$\;q_{rad}^{\prime \prime }\;$represents the radiation heat flux applied externally on the material, σ is the Stefan–Boltzmann constant, and $T_{s}$ and$\;T_{back}$ represent the temperatures of the material’s front and back surfaces, respectively. $T_{0}\;$refers to the initial ambient temperature,$\;P_{0}$ represents the atmospheric pressure, $h_{front}\;$and $h_{back}$ are the convective heat transfer coefficients for the front and back surfaces of the material, and ε represents the material’s emissivity.

Based on the heat transfer equation, decomposition equation, gas diffusion equation, and boundary conditions mentioned above, a mathematical model for the temperature-pressure coupling of glass fiber-reinforced phenolic resin material can be established.

## 3. Finite Element Implementation

### 3.1. User Subroutine Development 

Because the heat transfer module in the finite element software ABAQUS 2017 does not meet the temperature-pressure coupling analysis and calculation required in this paper, the UMATHT and USDFLD subroutine are developed to establish a mathematical model of temperature–pressure coupling analysis of polymer matrix composites and realize the coupling calculation of the heat transfer equation, Arrhenius equation, Darcy’s law, and ideal gas state equation.

The USDFLD subroutine is used to define and update the relevant field variables in the temperature–pressure coupled analysis model, ensuring that the data is promptly updated after each analysis step. The updated field variable data and other relevant data will be invoked in the next analysis step within the UMATHT subroutine, which was used to define the thermal constitutive behavior of materials and internal heat generation during the heat transfer process. It will be called at all material calculation points where the elements with thermal material behavior are defined. The COMMON BLOCK is the only implementation of global variables in the Fortran77 language. Variables within the same COMMON data block can be referenced between program units to achieve global variable sharing.

**Figure 1:**
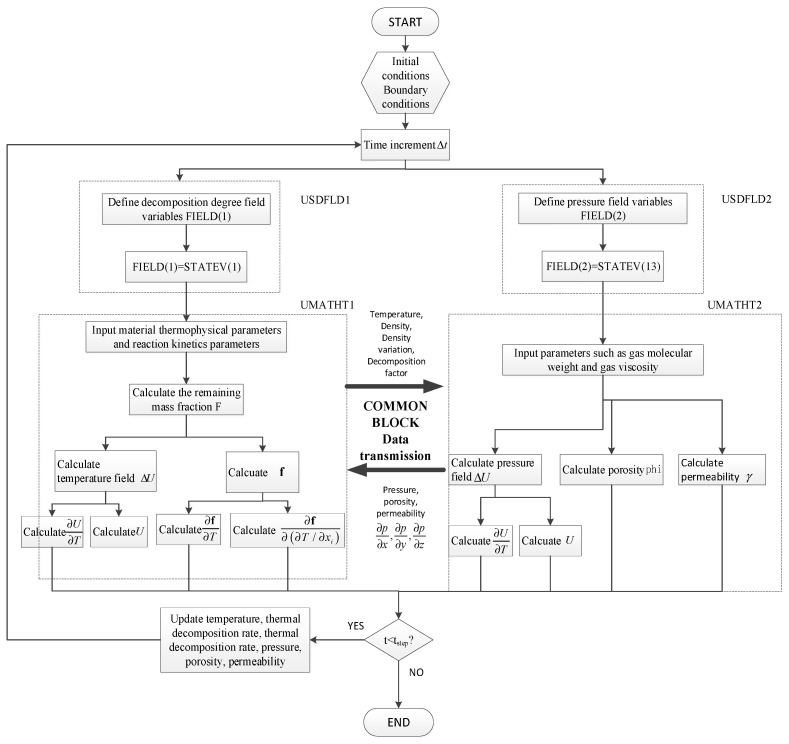
Flow chart of the temperature-pressure coupled analysis.

With the USDFLD and UMATHT interfaces as described above, the established mathematical model is compiled following the FORTRAN syntax rules. The communication between USDFLD and UMATHT is established through the STATEN state variable array, and the two UMATHT subroutines communicate with each other via the COMMON BLOCK module. All the programs are written in a. for file, and the compilation is done step by step in the order of USDFLD, UMATHT, and COMMON BLOCK. The flowchart of the subroutine calculation process is shown in Figure 1.

### 3.2. Overlapping Mesh Technique

**Figure 2:**
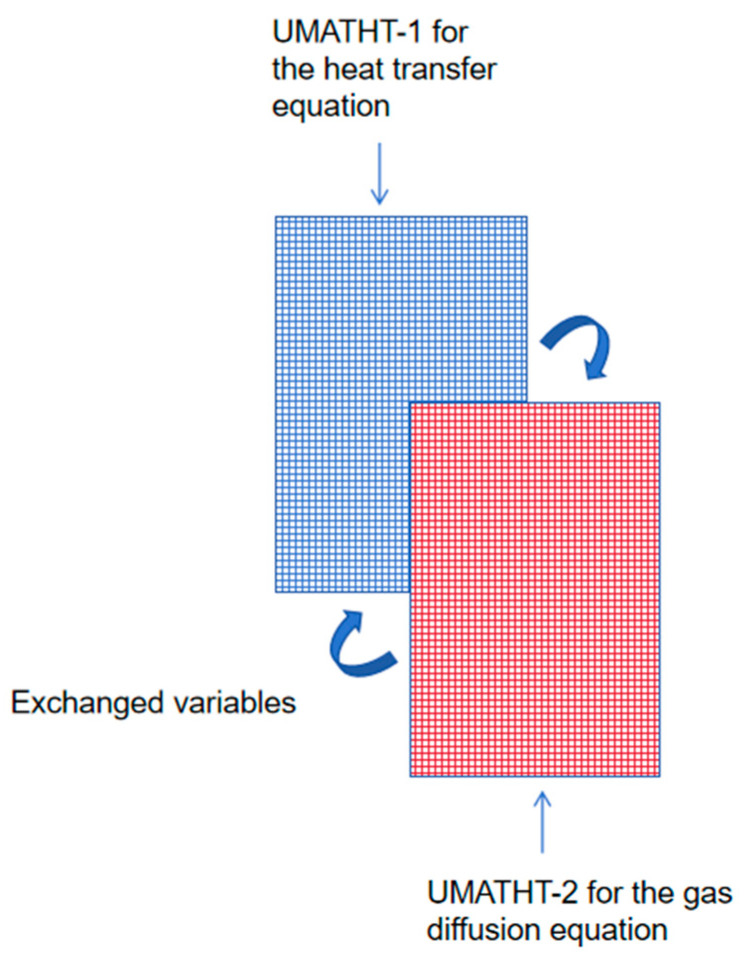
Schematic diagram of the overlapping mesh.

Due to the fact that heat transfer Equation (1) and gas diffusion Equation (6) have the same form of differential equation, two user subroutines—UMATHT-1 and UMATHT-2—are used to define the heat transfer equation and gas diffusion equation separately. Because one set of mesh can only apply to one UMATHT subroutine in the finite element analysis process, it is necessary to establish two sets of completely consistent mesh elements using an overlapping mesh technique, which is applied to the respective UMATHT subroutines. The schematic diagram is shown in Figure 2.

In the temperature–pressure coupled mathematical model, the temperature and pressure, as well as other relevant physical quantities, need to be transferred between the heat transfer equation and gas diffusion equation after each analysis step. Therefore, data storage and transfer are implemented through the COMMON BLOCK module in UMATHT-1 (heat transfer equation) and UMATHT-2 (gas diffusion equation). This allows both UMATHT subroutines to operate simultaneously in the two sets of mesh elements. The transfer and invocation of field variables is conducted following each analysis step, ensuring the complete coupling of temperature and pressure.

### 3.3. Experimental Description and Finite Element Model 

**Figure 3:**
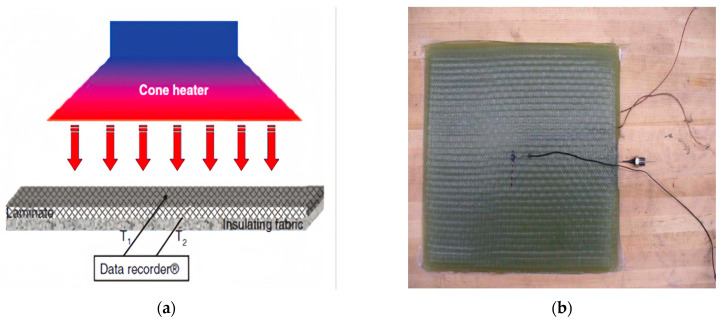
(**a**) Schematic diagram of test configuration (**b**) Sample with pressure and temperature probes [23].

Two different GFRP materials and their thermal response experiments were modeled and analyzed. The first material is glass fiber/vinyl ester composites, which consist of Vetrotex 324E fiberglass and decane 410–350 vinyl ester resin, with a resin content of 30% and a total of 20 layers of radial fibers. The curing agents include 0.06%, 4 pentanedione (retarder), 1.5% MEKP-925H (peroxide initiator), and 0.2% cobalt naphthenate (catalyst) [23]. The specimen, whose size is 100 mm × 100 mm × 12 mm, is fabricated using the vacuum-assisted resin transfer molding (VARTM) process. Probes for pressure measurement are embedded at specified locations within the specimen, and two K-type thermocouples are attached to the specimen surface for temperature measurement, as shown in Figure 3b. In the experiments, a cone calorimeter is used with a thermal load of 75 kW/m^2^ according to ASTM E 1354 [24]. Part-1 and Part-2 in the FE analysis are established based on the specimen, and the boundary conditions are set as T_0_ = 300 K and P_0_ = 0.101325 MPa. The mesh element type is DC3D8. Heat load is applied to the surface of Part-1, and the analysis step time is set to 2400 s. The parameters for the glass fiber/ethylene vinyl ester are shown in Table 1.

The second material is composed of Glass fiber/Phenolic (H41N), which contains 39.5% phenolic resin and 60.5% glass and talc fillers, and includes coupling agents and adhesives to ensure good adhesion. The size of the specimen is 100 mm × 100 mm with a thickness of 30mm. The initial temperature is 40 °C, and the initial pressure is 0.101325 MPa (1 atm). K-type thermocouples are implanted at central positions of 1 mm, 10 mm, and 29 mm in the specimen to measure temperature profiles. Subcutaneous injection tubes are inserted at positions of 6mm and 22.5 mm, sealed with ceramic adhesive to prevent gas leakage. A miniature strain gauge is attached to the free end of each tube to record the pressure generated during the decomposition process of the material. A cone calorimeter experiment was conducted under a heat flux of 279.7 kW/m^2^, following the ASTM E 1354 standard. Based on the model size, Part-1 and Part-2 in the FE analysis are established. A heat load is applied to the heated surface of Part-1, and initial temperature, initial pressure, and other environmental parameters are set. Both parts use the DC3D8 element type, and the computation time is set to 800 s, consistent with the heating time. The relevant parameters for the H41N material are shown in Table 2.

## 4. Results and Discussion

### 4.1. Glass Fiber Vinyl Ester 

**Figure 4:**
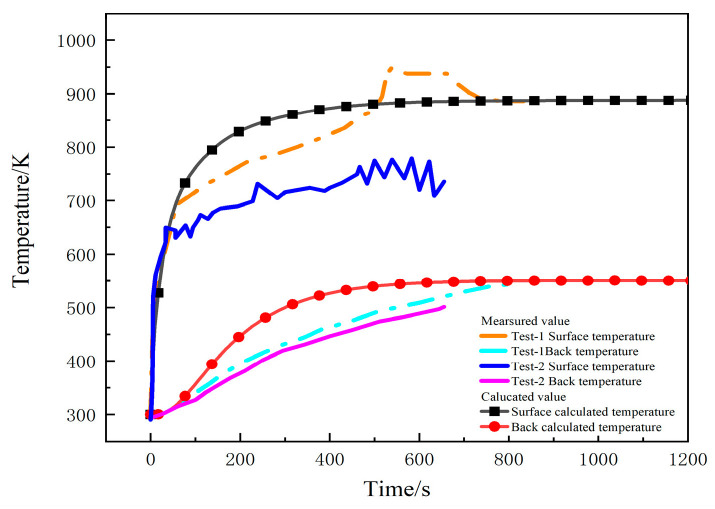
Comparison of the measured and calculated temperatures under 75 kW/m^2^ heat flux in two tests.

**Figure 5:**
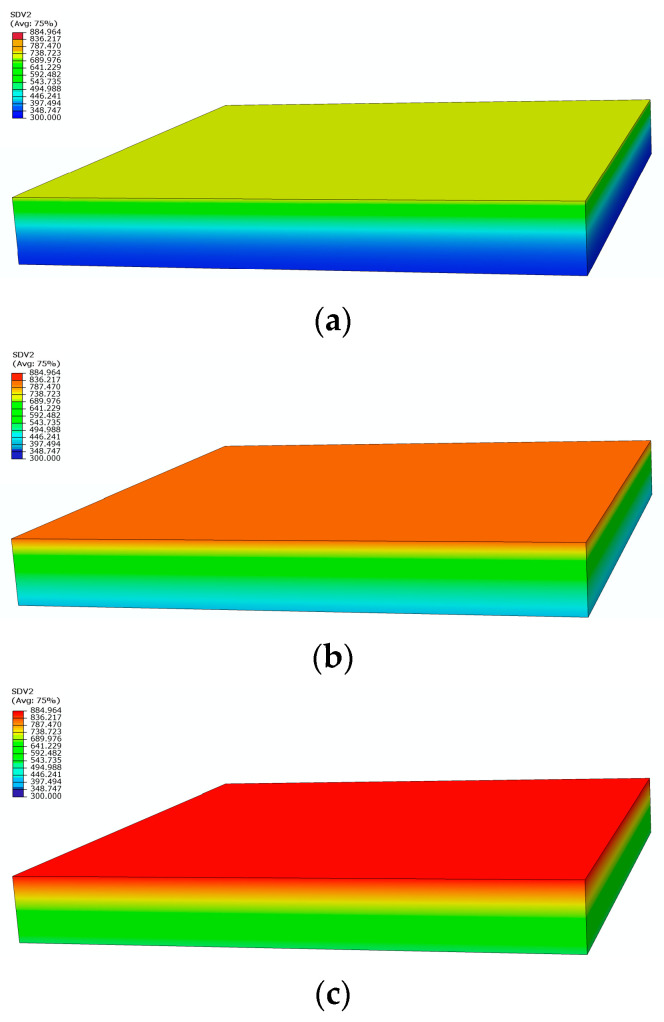
Temperature distribution contours at different moments: (**a**) 100 s; (**b**) 250 s; (**c**) 1200 s.

Figure 4 shows the comparison between the measured and calculated temperatures on the heating surface and the back under a heat flux of 75 kW/m^2^ in two tests. Due to the high temperatures during the second experiment, the thermocouples malfunctioned, resulting in us obtaining temperature data for only about 800 s [23]. The temperature curves calculated by the model are consistent with the measured curves, predicting the temperature trend, and finally reaching a dynamic equilibrium state. Figure 5 depicts the temperature contour maps of the sample at 100 s, 250 s, and 1200 s, respectively. Heat gradually transfers layer by layer along the thickness direction of sample, showing a gradient temperature distribution.

**Figure 6:**
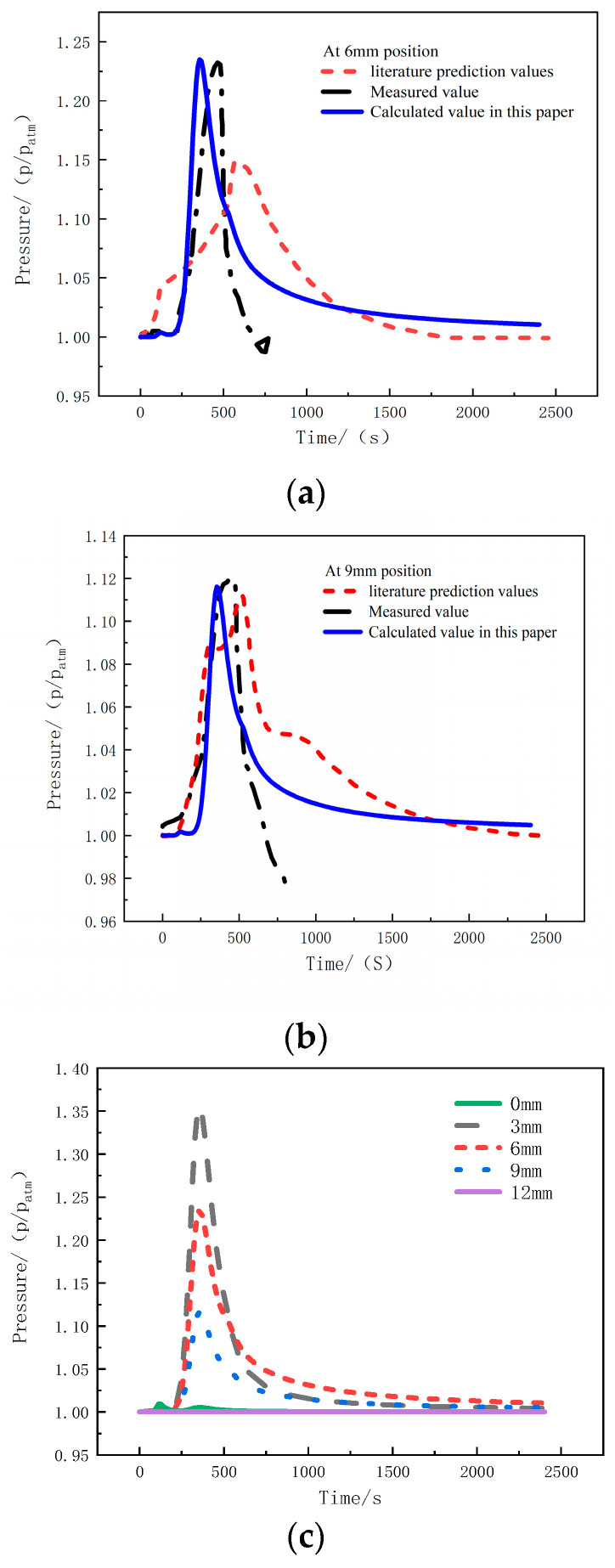
The pressure comparison under 75 kW/m^2^ heat flux at (**a**) 6 mm; (**b**) 9 mm; (**c**) multiple positions [23].

Figure 6a shows the comparison between the measured and predicted values of pressure at the 6 mm position in the thickness direction under a heat flux of 75 kW/m^2^. The pressure at this position rapidly increases around 250 s, and reaches a peak value at around 400 s, with a magnitude of 1.23 atm. Then, it decreases and eventually stabilizes at 1.01 atm, slightly higher than standard atmospheric pressure. Figure 6b presents the comparison of the measured and predicted pressure values at the 9 mm position under the same heat flux of 75 kW/m^2^. The pressure trend is similar to that of the 6mm position, but the peak pressure is relative lower—namely 1.12 atm—and the final pressure value is 1.005 atm. The predicted pressure values align well with the experimental measurements, but both pressure measurements are interrupted around 800 s, indicating the failure of the pressure probe and the K-type thermocouple simultaneously [23].

Figure 6c presents the pressure calculation curves at positions of 0 mm, 3 mm, 6 mm, 9 mm, and 12 mm for glass-fiber-reinforced polyethylene material under a heat flux of 75 kW/m^2^. At the 0 mm position, namely the heated surface, there is no significant change in pressure. The peak pressure at the 3 mm, 6 mm, and 9 mm positions decreases successively. Therefore, it can be observed that the closer the position is to the heat source, the higher the peak pressure. However, when the pressure begins to decrease and tends to be stable, the maximum pressure occurs at the 6mm position with a value of 1.01 atm, slightly higher than standard atmospheric pressure, which indicates that the escape of internal gas in the sample is relatively difficult than that closer to the surfaces.

**Figure 7:**
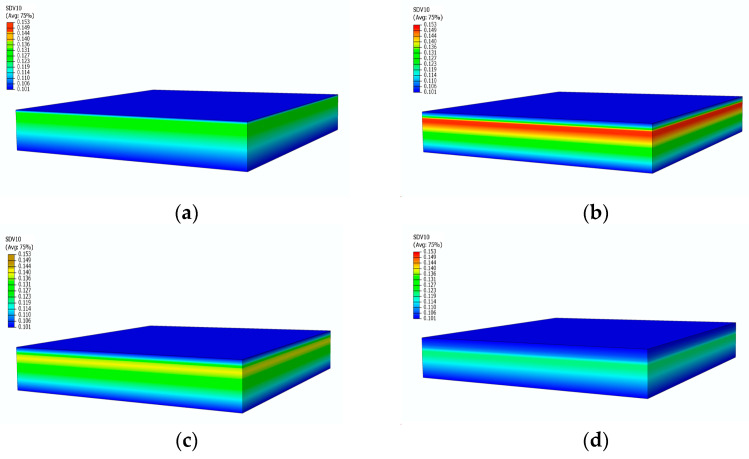
Pressure contours of glass fiber vinyl ester composite samples at different moments: (**a**) 100 s; (**b**) 480 s; (**c**) 550 s; (**d**) 2400 s.

Figure 7 shows the pressure distribution contours at different moments. Under a heat flux of 75 kW/m^2^, the temperature of the sample continues to rise. At 250 s, the surface temperature reaches 828.54 K, and the backside temperature reaches 444.91 K. Decomposition gas begins to generate internally, accompanied by the increase of pressure. At 480 s, the pressure reaches its peak at various positions. Subsequently, the porosity and permeability increase, and the gas starts to escape outward, leading to a decrease in pressure. However, the internal pressure ultimately remains slightly higher than the external atmospheric pressure.

By comparing the temperature and pressure curves calculated by the model with experimental measurements, the agreement of them indicates that the method developed in this article can be used to predict the temperature and pressure variations of glass-fiber-reinforced polyethylene under a one-sided heat flux environment.

**Figure 8:**
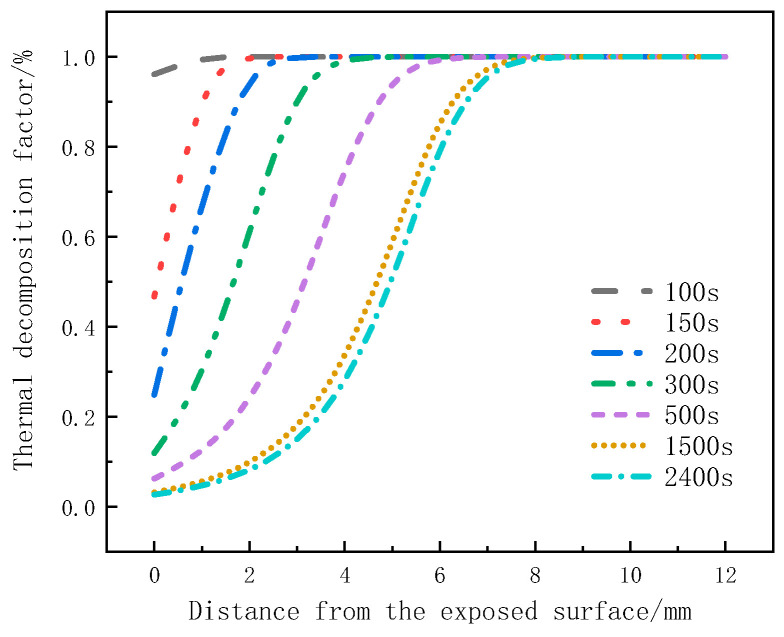
The thermal decomposition rate varies with thickness position at different moments.

Figure 8 illustrates the variation of the decomposition factor with thickness at different moments for glass fiber vinyl ester under a heat flux of 75 kW/m^2^. It can be seen that the decomposition factor of material at the surface layer starts to decrease at 100 s, indicating that material at this position is gradually undergoing pyrolysis. From 100 s to 150 s, the decomposition factor rapidly decreases, indicating intense pyrolysis. From 200 s to 500 s, the change in the decomposition factor slows down for the surface layer, while it rapidly increases for the 2mm to 4mm positions. From 1500 s to 2400 s, the decreasing trend of the decomposition factor becomes less pronounced, indicating a weaker pyrolysis intensity and slow decomposition. At 2400 s, the decomposition factor at around 8 mm for the glass fiber vinyl ester remains at 1, indicating that no pyrolysis reaction has occurred in the 8 mm to 12 mm region. Under the same pyrolysis time, for the surface layer, the decomposition factor decreases with thickness, and the change in the decomposition factor is significant, which can be used to determine the occurrence time of the pyrolysis reaction. At the same position, the decomposition factor decreases as the pyrolysis reaction progresses, and this change slows down with heating time.

**Figure 9:**
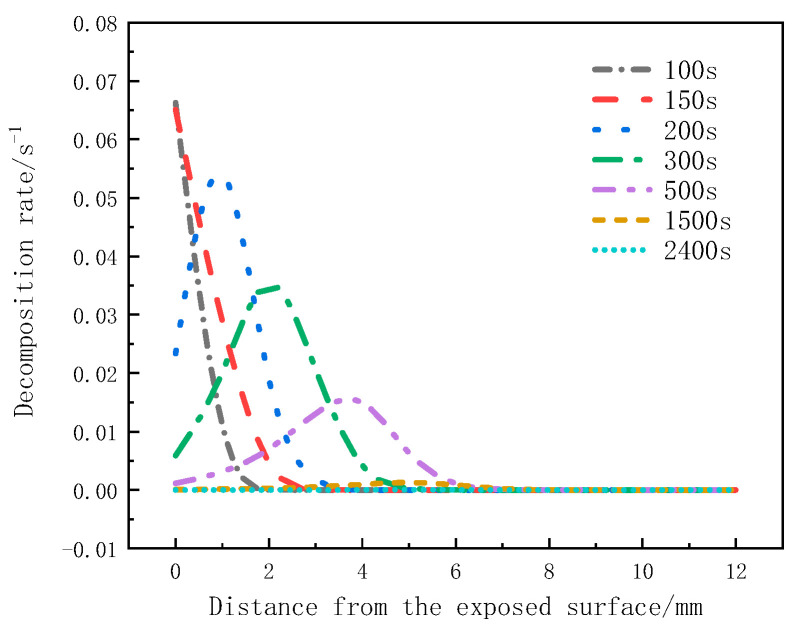
Decomposition rate varies with thickness at different moments.

Figure 9 shows the variation of the decomposition rate with thickness at different moments. The peak decomposition rates at 100 s and 150 s occur on the heated surface, but at 200 s, it is at the 1mm position. At 300 s and 500 s, it is at the 2 mm and 4 mm positions, respectively. There is no significant change in the decomposition rate at 1500 s and 2400 s. It can be observed that the peak decomposition rates at different moments move from the heated surface towards the back surface, and the peak decomposition rate decreases as the distance from the heat source increases.

**Figure 10:**
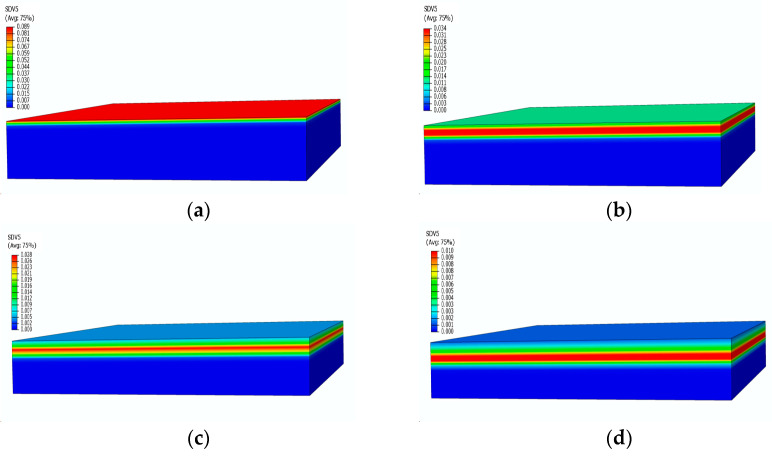
Decomposition rate distribution contours of glass fiber vinyl ester at different moments: (**a**) 100 s; (**b**) 200 s; (**c**) 500 s; (**d**) 2400 s.

Figure 10 presents the contours of the decomposition rate distribution at different moments. With the increase in temperature, the surface of the material starts to undergo pyrolysis, leading to an increase in the decomposition rate, reaching its peak in the strong pyrolysis zone, indicated by the red area in Figure 10a. Subsequently, the strong pyrolysis zone moves towards the back surface, and the peak decomposition rate begins to decrease. The decomposition rate then decreases as the pyrolysis reaction slows down, eventually forming carbonized material with a decomposition rate of 0. After 500 s, when the temperature reaches an equilibrium state, the strong decomposition zone is located at the 4 mm position, as shown in Figure 10c. At this point, the temperature no longer undergoes significant changes, and the pyrolysis intensity gradually decreases, as evident in the significantly decreased decomposition rates at 1500 s and 2400 s.

**Figure 11:**
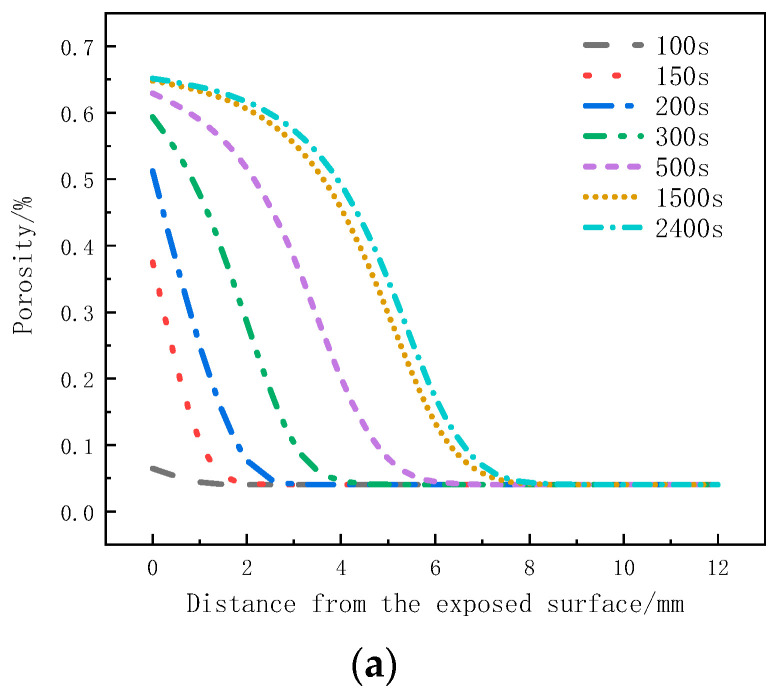

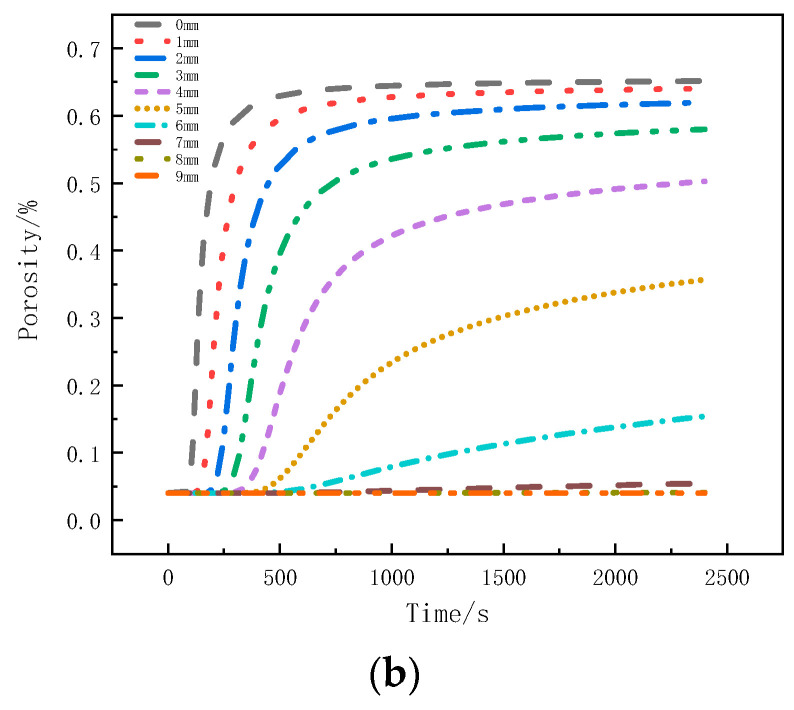
Porosity change curve: (**a**) Variation with thickness; (**b**) Variation with time.

Figure 11a shows the variation of the porosity with thickness at different moments. Within the initial 300 s, the pyrolysis reaction on the surface layer is more intense, resulting in the largest change in porosity. In the subsequent time, the pyrolysis reaction on the surface layer slows down, and the strong pyrolysis zone starts to move downwards. The porosity change at the following positions increases. At this time, the porosity at the 0 mm to 1mm position is already close to the porosity of coke, indicating the gradual formation of a carbonized region. After 500 s, material on the surface layer has completely pyrolyzed, forming coke, and the porosity no longer changes. The strong pyrolysis zone moves towards the back surface, and the porosity at the 0 mm to 6 mm positions gradually increases. From 1500 s to 2400 s, the change in porosity slows down. As the porosity of the sample is related to the decomposition factor, the weakening of the decomposition intensity at this moment also leads to less significant changes in porosity. 

Figure 11b presents the porosity variation with time at different positions. The regions closer to the heat source, within the 0mm to 4mm range, exhibit more pronounced decomposition, with the porosity increasing rapidly and reaching stability. The porosity at the 0mm position reaches 0.65, and as the depth increases, the peak porosity gradually decreases. The porosity at the 7 mm position is 0.06, while the porosity at the 8 mm position remains consistent with the initial porosity, indicating no change in porosity at that position.

**Figure 12:**
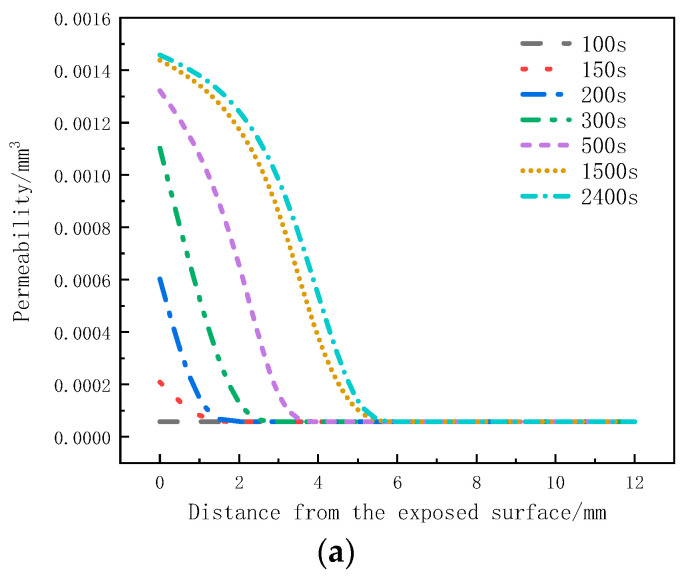

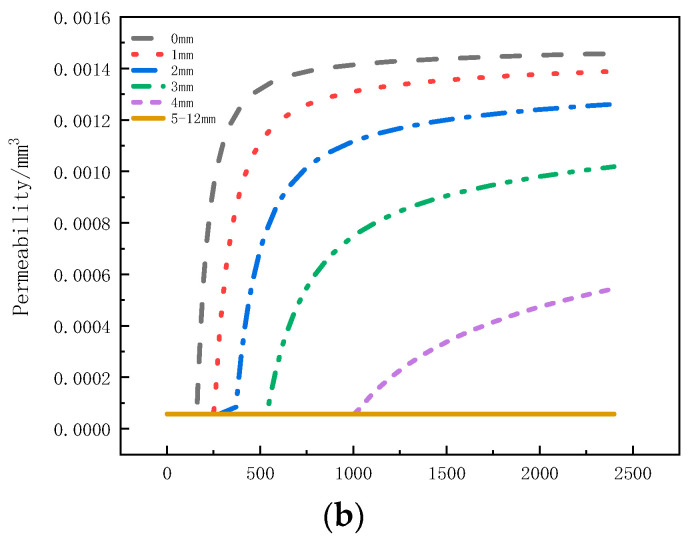
Permeability Variation: (**a**) with thickness; (**b**) with time.

Figure 12a illustrates the distribution of permeability in the thickness direction at different moments. Within the initial 100 s, the permeability remains unchanged. From 150 s to 500 s, there is a noticeable increase in permeability within the 0 mm to 4 mm region. From 500 s to 1500 s, the permeability in the 3.5 mm to 5 mm region starts to increase. However, in the time interval of 1500 s to 2400 s, the rate of increase in permeability at the 0 mm to 5 mm positions significantly slows down. Figure 12b presents the variation of permeability with time at different positions. It can be observed that the permeability of the sample within the 0 mm to 4 mm range increases with heating time and eventually reaches a stable state. The maximum permeability at the 0mm position reaches 1.46 × 10^−3^ mm^3^. Within this range, as the depth increases, the onset time for the increased permeability is delayed, and the maximum permeability decreases. The maximum permeability at the 4mm position is only 5.46 × 10^−4^ mm^3^. The increase in permeability leads to faster gas escape, which is one of the reasons for the decrease in internal pressure. The permeability remains unchanged within the 5 mm to 12 mm range.

Based on the comprehensive analysis of pressure, decomposition factor, permeability, and porosity, the following conclusions can be drawn. At the 3 mm position, the pressure rapidly decreases after reaching its peak. The decomposition rate shown in Figure 9 indicates that during this period, the intense pyrolysis zone moves towards the back, leading to a decrease in the decomposition rate and production of pyrolysis gas. Additionally, Figure 11b shows an increase in porosity. During the pressure decrease stage, the permeability at this position also gradually increases (as shown in Figure 12a). The combined effect of decreased decomposition rate, reduced pyrolysis gas production, increased porosity, and increased permeability contributes to the rapid pressure decrease after reaching its peak. At the 6 mm position, the pressure gradually decreases after reaching its peak. During this process, the permeability at this position remains unchanged (as shown in Figure 12b), but the decomposition factor in Figure 8 indicates pyrolysis has occurred, and Figure 11a shows an increase in porosity. Therefore, the main reason for the gradual pressure decreases after reaching its peak at this position is the increase in porosity. At the 9 mm position, both permeability and porosity remain unchanged, and the decomposition factor in Figure 8 also indicates no pyrolysis reaction. As a result, the reason for the initial increase and subsequent slow decrease in pressure at this position is likely due to the expansion of gas in the initial pores. However, due to the influence of the original permeability and porosity of the gas, the pressure starts to slowly decrease.

By comparing the pressure decrease rates at the three positions, it can be observed that material at the 3 mm position has the highest rate of decrease, which can be attributed to the combined effects of decomposition rate, permeability, and porosity. The pressure decrease trend at the 6 mm position slows down, and the final pressure is the highest. This is caused by the increase in porosity and the initial permeability. The pressure decrease at the 9 mm position is the slowest, primarily influenced by the initial permeability. Therefore, the increase in pressure is mainly influenced by high temperature and matrix pyrolysis, with the matrix pyrolysis being the main factor. The pressure decrease is determined by the weakening of the decomposition rate, permeability, and porosity.

### 4.2. Glass Fiber Phenolic

**Figure 13:**
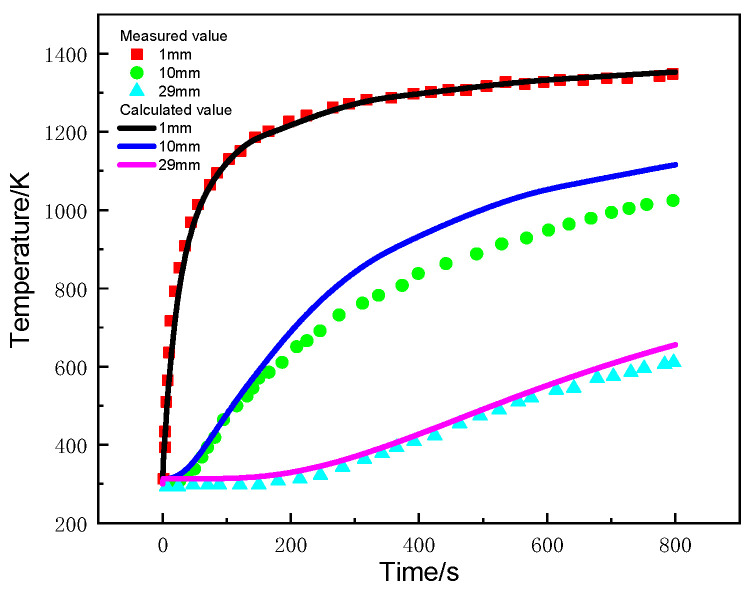
Curves of temperature changes with time at different positions.

Figure 13 compares the calculated and experimental temperature of glass-fiber-phenolic composites at positions of 1 mm, 10 mm, and 29 mm. The calculated values are in good agreement with the measured values, except that at the 10 mm position, which is slightly higher than the measured value. 

During the initial heating stage, the temperature of the sample at the 1 mm position increases rapidly. After 200 s, the temperature rise rate decreases, but pyrolysis temperature has already been reached and the thermal decomposition reaction initiated, absorbing heat and causing the temperature rise rate to decrease. Heat is transferred in the thickness direction of the material, and the temperature rise rate at the 10 mm and 29 mm positions decreases with thickness. The main reasons for this include carbonization of the material near the surface, which enhances the ability to dissipate heat through radiation, and diffusion of pyrolysis products to the heated surface, leading to heat loss and a decrease in the net heat flow into the interior of the material. 

**Figure 14:**
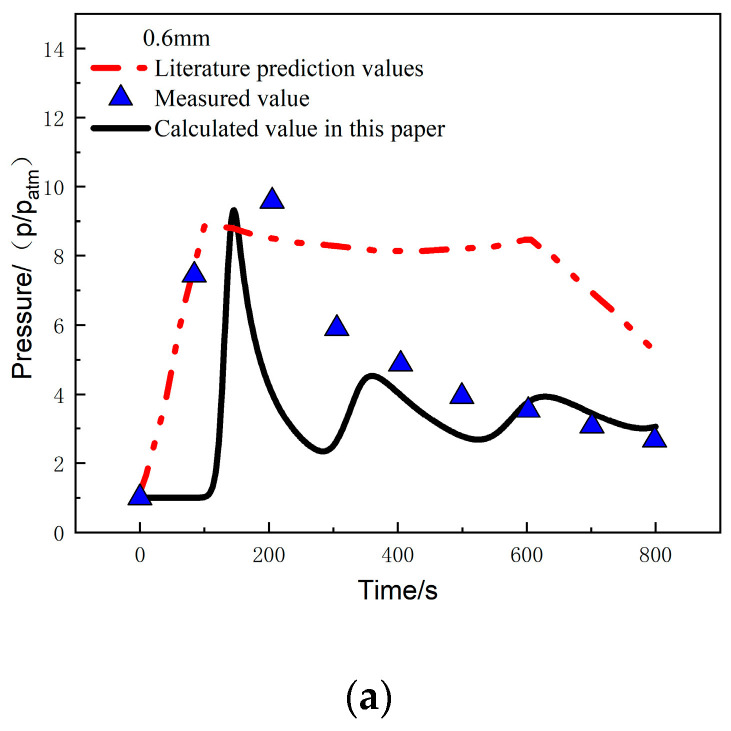

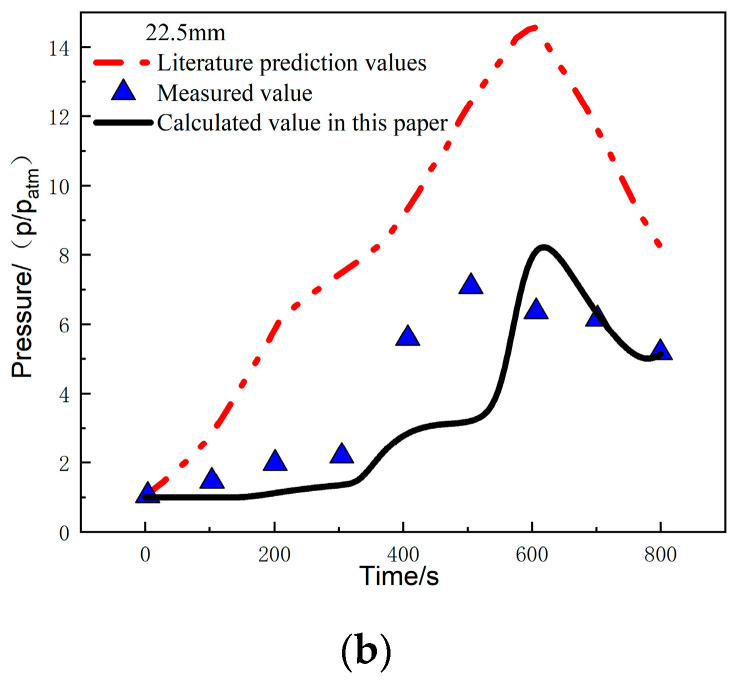
Pressure comparison at different positions: (**a**) 0.6 mm; (**b**) 22.5 mm [26].

Figure 14a compares the calculated and experimentally measured pressure values at the 0.6 mm position under a heat flux of 279.7 kW/m^2^. It can be observed that the pressure at this position rapidly increases at 100 s and reaches a peak pressure of 9.12 atm at 150 s. The error between the calculated and measured values in this study is 2.7%. After reaching the peak, the pressure starts to decrease, and from 400 s to 800 s, it shows good agreement with the measured values, with a pressure of 3.05 atm at 800 s. Figure 14b compares the calculated and experimentally measured pressure values at the 22.5 mm position. The trends of the two are consistent, and the pressure reaches its peak at around 620 s, with a peak pressure of 8.21 atm. The error between the calculated and measured values is 11.6%, and the pressure at 800 s is 5.1 atm.

The temperature-pressure coupling analysis method provides good agreement between the calculated temperature, pressure, and experimental measurements. The peak pressure and peak time are consistent with the experimental measurements, with errors much lower than the predicted results in reference [26]. This also indicates that the method in this study can be used to predict the temperature and internal pressure changes under a one-sided heat flux environment.

**Figure 15:**
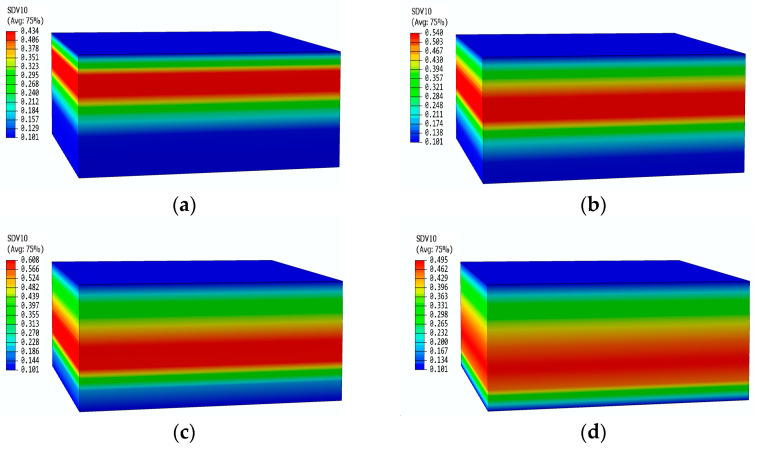
Pressure distribution contours of glass fiber phenolic material at different times: (**a**) 200 s; (**b**) 400 s; (**c**) 600 s; (**d**) 800 s.

Figure 15 shows the pressure contours of the glass fiber phenolic composite material at different moments. It can be observed that there is no change in pressure on the heated surface and the back surface. At 200 s, the pressure only increases within the range of 0–15 mm, with a peak pressure of 0.43 MPa. At 400 s, the peak pressure region shifts downward, with an increased peak pressure of 0.54 MPa, while the pressure near the heated surface starts to decrease. At 600 s, the area where the pressure increases expand further, and the peak pressure reaches 0.628 MPa. At 800 s, the peak pressure region moves further towards the back surface, with a peak pressure of 0.495 MPa. The pyrolysis region continuously moves towards the back surface, causing the peak pressure region to move accordingly. The decrease in decomposition rate results in a gradual decrease in peak pressure.

**Figure 16:**
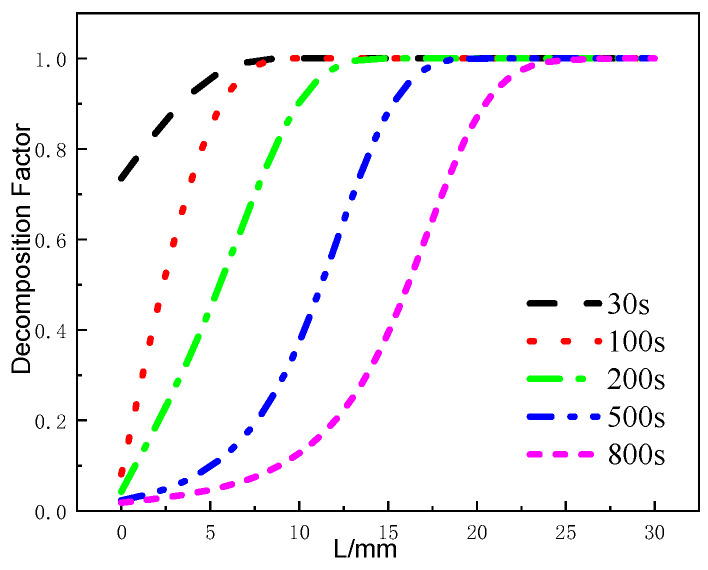
Decomposition factor curves at different moments.

Figure 16 presents the variation of the decomposition factor of the glass fiber phenolic composite material at different moments. At 30 s, when the temperature on the heated surface reaches 836 K, the decomposition factor at the 0mm position has already decreased to 0.72, indicating varying degrees of thermal decomposition within the 0–5mm range. At 100 s, the decomposition factor at the 0 mm position decreases to around 0.1, and the decomposition region still remains within the 0–5 mm range, with further decomposition occurring in this region. At 200 s, the decomposition region starts to move towards the back surface in the thickness direction, and gradual thermal decomposition reactions occur within the 5–12 mm range. From 500 s to 800 s, the variation range of the decomposition factor within the 0–5 mm range decreases, and the material within the 25–30 mm position ultimately remains undecomposed.

**Figure 17:**
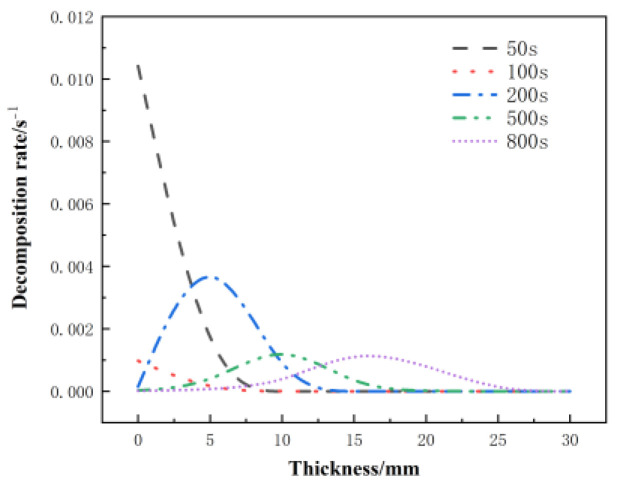
Variation of decomposition rate with thickness at different moments.

**Figure 18:**
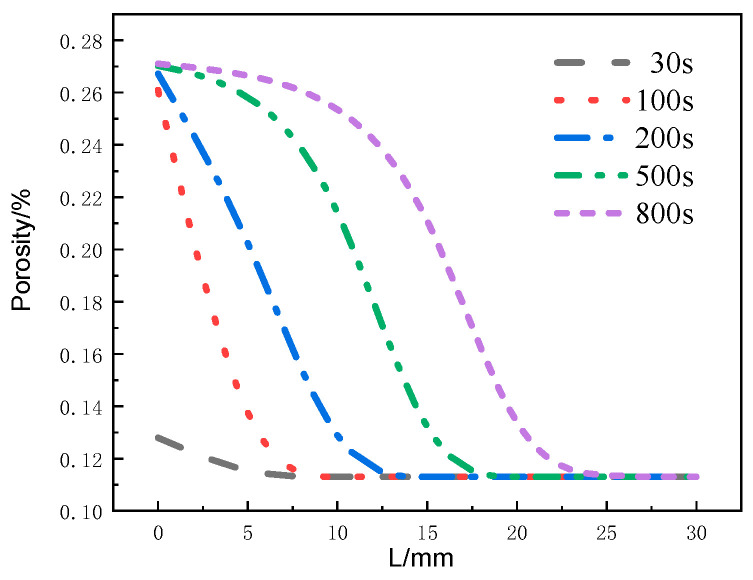
Variation of porosity with thickness at different moments.

Figure 17 shows the distribution of the decomposition rate with respect to thickness at different moments for the glass fiber phenolic composites. At 50 s, the decomposition rate is the highest at the 0 mm position and decreases with increasing thickness, with the decomposition rate at the 10 mm position dropping to zero. At 100 s, the decomposition rate on the heated surface further decreases. At 200 s, the heated surface has completely decomposed, with a decomposition rate of zero. Then, thermal decomposition reactions begin to occur, with the maximum decomposition rate observed at the 5 mm position. Subsequently, at 500 s and 800 s, the peak region of the decomposition rate gradually moves towards the back surface, and the peak value of the decomposition rate begins to decrease. Eventually, the pyrolysis region reaches the 25 mm position. Figure 18 presents the distribution of the porosity rate with respect to thickness at different moments for the glass fiber phenolic composites. It can be observed that at 30 s, the material on the heated surface has undergone decomposition, with the porosity rate reaching 0.13. At 100 s, the porosity rate reaches 0.26, indicating a rapid increase in porosity rate in the region near the heated surface within the first 100 s, as intense thermal decomposition in this area has already occurred. The porosity rate is increased in the 0–10 mm range and decreases with increasing thickness. The porosity rate in other positions remains at the initial level. As the heating time progresses, the porosity rate in the 10–25 mm range gradually increases. At 800 s, the porosity rate in the 0–5 mm position approaches that of the carbonized material, while the porosity rate in the 25–30 mm range remains unchanged.

**Figure 19:**
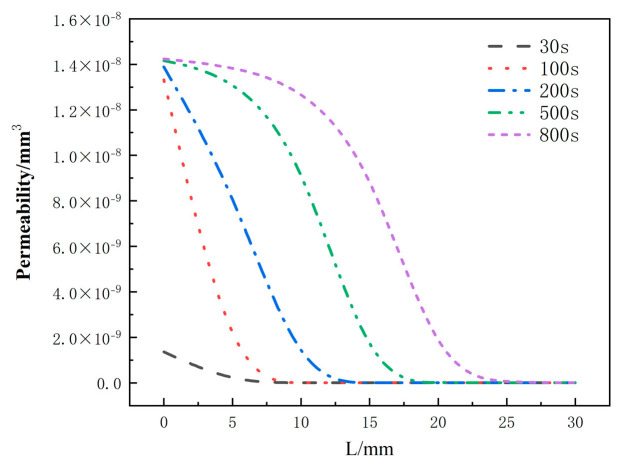
Variation of permeability with thickness at different moments.

Figure 19 depicts the distribution of the permeability rate with respect to thickness at different moments for the glass fiber phenolic composites. It can be observed that the permeability rate follows a similar trend as the porosity rate, with an increase in permeability rate from 0 mm to 25 mm as heating time progresses, indicating easier gas escape. At the same moment, the permeability rate decreases with increasing thickness. At the same position, the permeability rate increases with heating time, and the increasing trend slows down when the permeability rate approaches that of carbonized material.

By analyzing the variations of decomposition rate, porosity rate, and permeability rate, it can be concluded that the change in pressure is due to the following reasons. At the 0.6 mm position, the temperature of the material rapidly increases and triggers a thermal decomposition reaction, resulting in the generation of thermal decomposition gases, which leads to an increase in pressure and reaches a peak. From Figure 7, it can be found that the decomposition already occurred at the 0.6 mm position at 100 s, but Figure 4a shows that the pressure at this position did not change, indicating a significant lag in pressure generation compared to the decomposition process. As the thermal decomposition rate at the 0.6 mm position gradually decreases, the generation of thermal decomposition products decreases, causing the pressure to start decreasing. However, at this point, the porosity rate and permeability rate increase (as shown in Figure 8 and Figure 9), making it easier for gas to escape.

At the 22.5 mm position, the increase in pressure can be divided into two stages. From 200 s to 500 s, the material decomposition rate remains unchanged (as shown in Figure 7), and the porosity rate and permeability rate also remain unchanged (as shown in Figure 8 and Figure 9). However, the temperature at this position continues to rise due to heat conduction (as shown in Figure 3). Therefore, during this stage, the temperature rise leads to a slow increase in internal pressure within the material. In the stage from 500 s to 620 s, the temperature at this position continues to rise, and a thermal decomposition reaction occurs, resulting in the generation of thermal decomposition gases, causing the pressure to increase rapidly. From 620 s to 800 s, the increase in porosity rate and permeability rate at this position (as shown in Figure 8 and Figure 9) causes the pressure to rapidly decrease.

In conclusion, through the analysis of the variations in decomposition rate, porosity rate, and permeability rate, it can be determined that the increase in pressure is caused by the rapid increase in temperature at the 0.6mm position, triggering a thermal decomposition reaction and generating thermal decomposition gases. The pressure increase is delayed compared to the decomposition process, as evident from Figure 7 at 100 s. As the thermal decomposition rate at the 0.6 mm position gradually decreases, the generation of thermal decomposition products decreases, leading to a decrease in pressure. However, at this point, the increase in porosity rate and permeability rate makes the escape of gas easier. At the 22.5 mm position, the increase in pressure occurs in two stages. From 200 s to 500 s, the material decomposition rate, porosity rate, and permeability rate remain unchanged, while the temperature at this position continues to rise due to heat conduction. Therefore, during this stage, the pressure slowly increases due to the temperature rise. At the 500 s to 620 s stage, the temperature continues to rise at this position, and a thermal decomposition reaction occurs, generating thermal decomposition gases, causing the pressure to increase rapidly. From 620 s to 800 s, the increase in porosity rate and permeability rate at this position causes the pressure to rapidly decrease.

## 5. Conclusions

1. A pressure prediction model is established for GFRP composites using heat transfer equations, decomposition equations, gas diffusion equations, the three-dimensional Darcy’s law, and the ideal gas state equation, considering polymer pyrolysis, gas diffusion, porosity, gas permeability, and gas viscosity changes. Based on the UMATHT and USDFLD user subroutines developed, the coupling calculation of temperature and internal pressure is realized using the overlapping mesh method. Therefore, a pressure prediction method for GFRP materials is established, and the predicted values are in good agreement with experimental measurements. This method can provide a calculation method for internal pressure in the thermal damage research of GFRP composites in fire.
2. Under the unilateral thermal radiation, GFRP composites undergo thermal decomposition, and the decomposition region gradually moves from the surface towards the interior of the material. The decomposition factor within the decomposition region starts to decrease, and the decomposition rate increases. Additionally, the porosity and permeability increase with heating time and eventually stabilize. As the depth increases, the rate of decrease in the decomposition factor slows down, and the peak of decomposition rate decreases. The starting time of the increase in porosity and permeability is delayed, and the maximum values decrease sequentially. The decomposition factor, decomposition rate, porosity, and permeability in the region near the back of the specimen remain unchanged.
3. The heating and back surfaces of the specimens of GFRP are exposed in the air environment, so the pressure will not change significantly. However, inter-pressure within the material surged due to the accumulation of thermal decomposition gases, and reached a peak value. After that, the pressure begins to decrease, but the factors affecting the pressure decrease vary at different positions. The pressure closest to the heating surface is influenced by the combined effects of decomposition rate, permeability, and porosity, while the pressure far away from the heating surface is only affected by the initial permeability. The pressure in the intermediate region may be influenced by both increased porosity and initial permeability.

## Figures and Tables

**Table 1:** Parameters of glass fiber/vinyl ester [25].

Parameters	Numerical Expression
Initial material density $\rho_{v}$/kg·m^−3^	1683
Final material density $\rho_{char}$/kg·m^−3^	1235
Thermal conductivity of initial material $K_{vi}$/W·m^−1^·K^−1^	4.405 × 10^−5^ × T + 0.512
Thermal conductivity of final material $K_{di}$/W·m^−1^·K^−1^	1.873 × 10^−4^ × T + 0.285
Specific heat capacity of initial material $C_{pv}$/J·kg^−1^·K^−1^	0.0452 × T + 1080
Specific heat capacity of final material $C_{pd}$/J·kg^−1^·K^−1^	0.259 × T + 1041
Gas specific heat capacity/J·kg^−1^·K^−1^	−91.1 + 4.4007 × T − 1.729710^−3^ × T^2^
Activation energy E/J·mol^−1^	3.62 × 10^5^
Reaction order n	4.6
Pre-reference factor A/1·s^−1^	5.0 × 10^28^
Decomposition heat Q/kJ·kg^−1^	−2 × 10^8^
Gas molecular weight/kg·mol^−1^	50 × 10^−3^
Porosity φ	0.668189 − 0.627953 × F
Gas viscosity/MPa·s	(1.2 × 10^−5^ + 1.5 × 10^−10^ × T) × 10^−6^
Permeability/mm^3^	1.56 × 10^−2^ − 3.99 × 10^−2^ × F, (F ≤ 0.39) 5.72 × 10^−5^, (F > 0.39)

**Table 2:** Material properties for H41N [26].

Parameters	Numerical Expression
Initial material density $\rho_{v}$/kg·m^−3^	2040.6
Final material density $\rho_{char}$/kg·m^−3^	1764
Thermal conductivity of initial material $K_{vi}$/W·m^−1^·K^−1^	2.76 × 10^−4^ × T + 0.8
Thermal conductivity of final material/$K_{di}$W·m^−1^·K^−1^	8.42 × 10^−4^ × T + 0.96
Specific heat capacity of initial material $C_{pv}$/J·kg^−1^·K^−1^	1.05 + 9.76 × 10^−4^ × T
Specific heat capacity of final material $C_{pd}$/J·kg^−1^·K^−1^	0.88 + 7.60 × 10^−4^ × T
Gas specific heat capacity/J·kg^−1^·K^−1^	2.394 + 1.05 × 10^−3^ × T
Activation energy E/J·mol^−1^	3.54 × 10^5^
Reaction order n	6
Pre-reference factor A/1·s^−1^	8.17 × 10^18^
Decomposition heat Q/kJ·kg^−1^	−234 × 10^5^
Gas molecular weight/kg·mol^−1^	18.35 × 10^−3^
Porosity φ	0.113 × F + 0.274 × (1 − F)
Gas viscosity/MPa·s	(1.48 × 10^−5^ + 2.5 × 10^−8^ × T) × 10^−6^
Permeability/mm^3^	6.18 × 10^−12^ × F + 4.85 × 10^−9^ × (1 − F)

## Data Availability

Data are contained within the article.
